# High sensitivity C-reactive protein and glycated hemoglobin levels as dominant predictors of all-cause dementia: a nationwide population-based cohort study

**DOI:** 10.1186/s12979-022-00265-0

**Published:** 2022-02-16

**Authors:** Yen-Chun Fan, Chia-Chi Chou, Bagas Suryo Bintoro, Kuo-Liong Chien, Chyi-Huey Bai

**Affiliations:** 1 School of Public Health, College of Public Health, Taipei Medical University, Taipei, Taiwan; 2 Institute of Epidemiology and Preventive Medicine, National Taiwan University, Taipei, Taiwan; 3 School of Medicine, Chang Gung University, Taoyuan, Taiwan; 4 Department of Internal Medicine, Chang Gung Memorial Hospital, Keelung, Taiwan; 5 Department of Health Behavior, Environment, and Social Medicine, Faculty of Medicine, Public Health, and Nursing, Universitas Gadjah Mada, Yogyakarta, Indonesia; 6 Center of Health Behavior and Promotion, Faculty of Medicine, Public Health and Nursing, Universitas Gadjah Mada, Yogyakarta, Indonesia; 7 Institute of Preventive Medicine, College of Public Health, National Taiwan University, Taipei, Taiwan; 8 Department of Internal Medicine, National Taiwan University Hospital, Taipei, Taiwan; 9 Department of Public Health, College of Medicine, Taipei Medical University, 250 Wu-Hsing Street, Taipei, 11031 Taiwan; 10 Nutrition Research Center, Taipei Medical University Hospital, Taipei, Taiwan

**Keywords:** Hs-CRP, HbA1c, Biomarker, Combined effect, Dementia

## Abstract

**Background:**

Chronic inflammation might play a major role in the pathogenesis linking diabetes mellitus (DM) to cognition. In addition, DM might be the main driver of dementia risk. The purpose of the present study was to evaluate whether inflammation, glycation, or both are associated with the risk of developing all-cause dementia (ACD).

**Methods:**

A nationwide population-based cohort study was conducted with 4113 participants. The data were obtained from the Taiwanese Survey on Prevalence of Hypertension, Hyperglycemia, and Hyperlipidemia (TwSHHH) in 2007, which was linked with the Taiwan National Health Insurance Research Database (NHIRD). The markers of inflammation, expressed as hs-CRP, and glycation, presented as HbA1c, were measured. High levels of hs-CRP and HbA1c were defined as values greater than or equal to the 66th percentile. Developed ACD was identified based on the International Classification of Diseases, Ninth Revision, Clinical Modification (ICD-9-CM) codes.

**Results:**

During 32,926.90 person-years, 106 individuals developed ACD in up to 8 years of follow-up. The study participants were separated into four categories by the top tertiles of hs-CRP and HbA1c based on the 66th percentile: high levels of both hs-CRP and HbA1c, only high levels of hs-CRP, only high levels of HbA1c, and non-high levels of hs-CRP nor HbA1c. Those who with a high level of only hs-CRP had the higher hazard for developing ACD (adjusted HR = 2.58; 95% CI = 1.29 ~ 5.17; *P* = 0.007), followed by the group with a high level of only HbA1c (adjusted HR = 2.52; 95% CI = 1.34 ~ 4.74; *P* = 0.004) and the group with high levels of both hs-CRP and HbA1c (adjusted HR = 2.36; 95% CI = 1.20 ~ 4.62; *P* = 0.012). Among those aged less than 65 years, hs-CRP was the only significant predictor of ACD risk (*P* = 0.046), whereas it did not yield any significant result in the elderly.

**Conclusions:**

A higher risk of developing ACD was found not only in patients with high levels of inflammation but also high levels of glycated hemoglobin. Future studies should focus on the clinical implementation of hs-CRP or HbA1c to monitor cognitive deficits.

## Background

The etiology of neurodegenerative diseases is multifactorial and is attributable to several major risk factors, [1] including hypertension, diabetes mellitus (DM), high cholesterol, obesity, physical inactivity, smoking, and depression [2–4]. These predictors contributing to the development of dementia could be modified through healthy lifestyle behaviors [5]. Therefore, it is crucial to discover potential biomarkers for the early development and progression of cognitive impairment [6].

Chronic inflammation might play a major role in the pathogenesis of type 2 DM to Alzheimer’s disease (AD) [7]. The co-morbid conditions related to diabetes, such as obesity, high cholesterol, and hypertension, were negatively associated with brain function [8]. In addition, the relationship between increased systemic inflammation and cognitive dysfunction is thought to result from physical inactivity and cigarette smoking [9, 10]. In addition, anti-inflammatory treatments could reduce dementia risk among people with depressive disorder [11]. Furthermore, chronic low-grade inflammation, measured using levels of high-sensitivity C-reactive protein (hs-CRP), was associated with early stage β-amyloid accumulation, resulting in neuroinflammation in brain regions [12].

Diabetes mellitus was found to be the main driver of cardiovascular risk factors and the risk of dementia due to increased insulin resistance (IR), which is influenced by diabetes [13]. The marker of glycated hemoglobin (HbA1c) might be clinically useful as a surrogate for identifying the presence of both insulin resistance and dysglycemia, [14] for which the possible biological pathogenesis might be that chronic hyperglycemia plays a key role in linking diabetes and memory decline, likely through microvascular injury [15]. Nevertheless, although HbA1c is a surrogate biomarker for detecting insulin resistance, it is suggested to be used in combination with other biomarkers [16].

The association between hyperglycemia and AD via tau hyperphosphorylation has been demonstrated [17]. In addition, inflammation is regarded as a major driver of IR in AD, impairing the blood–brain barrier [18]. However, the damaging consequences of IR have different pathomechanisms in DM and AD [19]. Moreover, previous studies have shown the joint effect of inflammation and glycation on cardiovascular diseases, such as coronary artery diseases, [20] cardiovascular risks, [21] and advanced subclinical carotid atherosclerosis progression, [22] which share similar pathogenic features that contribute to cerebral white matter hyperintensities, atherogenesis, and focal dysregulation in cerebrovascular flow in the hippocampus, leading to cognitive decline [23, 24]. However, there is still no evidence that illustrates the impact of the combination of inflammation and glycation on the risk of developing dementia.

Therefore, the purpose of the present study was to determine whether there is a possible impact from inflammation, glycation, or both on the risk of developing all-cause dementia (ACD) during eight years of follow-up in a nationwide population-based study sample.

## Methods

### Study design and data sources

This study was a cohort study. Data were obtained from the Taiwanese Survey on Prevalence of Hypertension, Hyperglycemia, and Hyperlipidemia (TwSHHH) in 2002. A second follow-up of the TwSHHH was carried out in 2007, encompassing a longitudinal study with a nationally representative sample. It was then linked with the Taiwan National Health Insurance Research Database (NHIRD) between 2001 and 2015 to identify information on ACD diagnoses. Moreover, age-subgroup analyses were conducted to evaluate the effect of inflammation, glycation, or both on ACD risk. The participants’ characteristics and the research design in recruitment regarding the TwSHHH and NHIRD were described in detail previously and maintained by the Health and Welfare Data Science Center, Ministry of Health and Welfare (HWDC, MOHW) [25, 26]. This study was reviewed and approved by the Joint Institutional Review Board at Taipei Medical University.

### Study sample

**Fig. 1:**
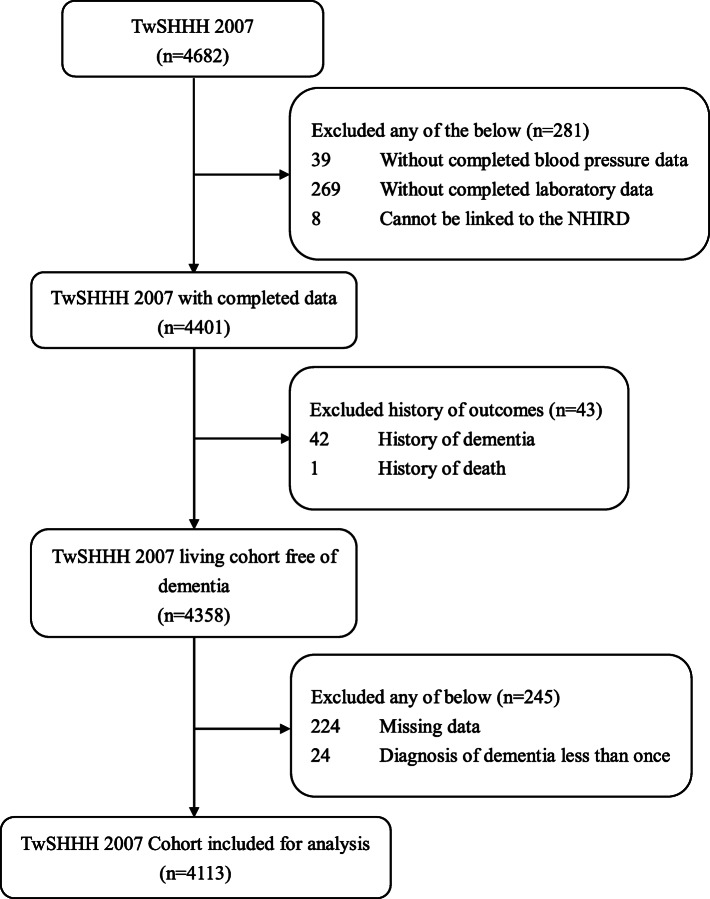
Flow chart of patient selection

Baseline participants in the cohort study were from the TwSHHH in 2007, with a total of 4682 samples aged 21–99 years. Those without complete data on blood pressure and biochemical laboratory data and those who could not be linked to the NHIRD were excluded (*n* = 281). Additionally, we excluded subjects with a history of ACD (*n* = 42) or who died (*n* = 1) before 2007. Moreover, in order to reduce the bias and increase the validity of the diagnosis, data sources with missing data on age, sex, laboratory measurements, lifestyle, and comorbidities, and participants with only a single diagnosis of ACD were also excluded (*n* = 245). Finally, 4113 samples were included in the analysis (Fig. 1).

### Definition of high levels of inflammation (hs-CRP) and glycation (HbA1c)

The subjects’ hs-CRP and HbA1c levels were examined during the second TwSHHH visit in 2007. The biomarker of inflammation, represented by hs-CRP, was measured using the particle-enhanced immunoturbidmetric principle. Glycation, represented by HbA1c, was assessed using high-performance liquid chromatography. The specimens were collected by trained technicians. All measurements, derived from 5% duplicated blood samples, were obtained with blinded quality control specimens in the central laboratory.

The cut-off points of high levels of inflammation and glycemic control were defined as values greater than or equal to the 66th percentile from the final sample. Therefore, the study participants were separated into three categories according to the top tertiles of hs-CRP and HbA1c levels, including both hs-CRP and HbA1c levels above the 66th percentile, either hs-CRP levels or HbA1c levels above the 66th percentile, and neither hs-CRP nor HbA1c levels above the 66th percentile. Additionally, participants with either hs-CRP or HbA1c levels above the 66th percentile were then divided into two groups: only hs-CRP above the 66th percentile and only HbA1c above the 66th percentile.

### Study outcome

The endpoint of ACD in this study was identified in the NHIRD according to the International Classification of Diseases, Ninth Revision, Clinical Modification (ICD-9-CM) codes. A diagnosis of ACD was defined as senile dementia, uncomplicated dementia (290.0), presenile dementia (290.1x), senile dementia with delusional or depressive features (290.2x), senile dementia with delirium (290.3), arteriosclerotic dementia (290.4x), dementia in conditions classified elsewhere (294.1), AD (331.0), Pick’s disease (331.1), and senile degeneration of the brain (331.2).

### Covariables

The covariates in this study were age, sex, and associated comorbidities that might affect the relationship between hs-CRP and HbA1c levels and the risk of ACD. Baseline demographic characteristics, including age and sex, were defined using the questionnaire. Laboratory data regarding systolic blood pressure, diastolic blood pressure, glucose, total cholesterol, triglycerides, and body mass index were obtained from the TwSHHH in 2007. However, the TwSHHH did not identify the illnesses associated with inflammatory disorders and infections. Therefore, we used the NHIRD to determine the variables of chronic infection or inflammation (ICD-9-CM 042–044, 010–018, and 090–099). In addition, these covariables were adjusted using statistical models.

### Statistical analysis

Statistical analysis system software (SAS System for Windows, version. 9.4; SAS Institute, Cary, NC, USA) was used to perform all statistical analyses. Those with only one ACD diagnosis were excluded to ensure the validity of the diagnosis. In this study, the index date to explore the joint effect of hs-CRP and HbA1C on ACD risk was set as the date of the second TwSHHH visit in 2007. In the final study sample, each subject was tracked from the index date to whichever came first: the development of ACD, death, or the end of 2015 (year).

Continuous and dichotomous variables were expressed as the mean ± standard deviation (SD) and as numbers with percentages, respectively. The comparison of the differences in the distributions of the demographic characteristics among the 4 groups was performed using the Chi-squared test and Kruskal-Wallis H test. Moreover, the risk of ACD was examined using a Cox proportional hazard regression model with hazard ratios (HRs) and 95% confidence intervals (CIs). In addition, a multivariate Cox proportional hazard regression was used to explore relationships between the combination of hs-CRP and HbA1c levels with ACD risk after adjusting for potential confounders such as age, sex, systolic blood pressure (SBP), diastolic blood pressure (DBP), glucose, total cholesterol, triglycerides, body mass index (BMI), exercise status, smoking status, alcohol consumption, heart disease, stroke, and chronic infection or inflammation. In all of the Cox models, the proportional hazard assumptions were not violated.

Furthermore, a subgroup analysis was carried out to investigate the impact of the joint effect of hs-CRP and HbA1c with the endpoints, including the primary endpoint of ACD among those without DM, those aged less than 65 years based on their age at baseline, and those aged 65 years and older; and secondary endpoints of AD and vascular dementia among the total sample, which tested the consistency of the results. Statistical significance was set at *P* < 0.05.

## Results

A total of 4113 individuals aged 21–99 years were enrolled, with a mean age of 47.11 years; 47% were male, and the participants had mean hs-CRP and hbA1c values of 0.22 mg/dL and 5.62%, respectively. During 32,926.90 person-years, 106 persons developed ACD in up to eight years of follow-up. These participants were classified into four categories, including both hs-CRP and HbA1c levels above the 66th percentile (756 subjects), only hs-CRP levels above the 66th percentile (682 subjects), only HbA1c levels above the 66th percentile (772 subjects), and neither hs-CRP nor HbA1c levels above the 66th percentile (1903 subjects).

The distributions of all demographic characteristics, except for lifestyle factors in alcohol consumption (*P* = 0.110) and comorbidities in chronic infection or inflammation (*P* = 0.147), differed significantly according to the status of the combination of hs-CRP and HbA1c. The mean age, systolic blood pressure, diastolic blood pressure, glucose, total cholesterol, triglycerides, and body mass index (all *P* < 0.001) of the participants and the proportion of men (*P* = 0.002), smoking behavior (*P* < 0.001), heart disease (*P* < 0.001), and stroke (*P* < 0.001) were all higher among those with high levels of both hs-CRP and HbA1c, as presented in Table 1.

**Table 1:** Distributions of demographic characteristics according to high-sensitivity C-reactive protein (hs-CRP) and glycated hemoglobin (HbA1c) levels

	Combined effect of hs-CRP and HbA1c ^†^				*P* value ^*^
	Both at high levels (*n* = 756)	High level of only hs-CRP (*n* = 682)	High level of only HbA1c (*n* = 772)	Neither at a high level (*n* = 1903)	
Age, mean (SD), years	55.68 (14.72)	45.44 (15.54)	54.79 (13.64)	41.18 (14.21)	< 0.001
Systolic blood pressure, mean (SD), mmHg	131.33 (18.99)	120.73 (17.64)	124.46 (16.77)	115.1 (16.89)	< 0.001
Diastolic blood pressure, mean (SD), mmHg	80.18 (11.38)	76.36 (11.42)	76.97 (10.54)	73.14 (11.05)	< 0.001
Glucose, mean (SD), mg/dL	109.36 (41.75)	85.48 (8.43)	102.82 (37.91)	83.33 (7.53)	< 0.001
Total cholesterol, mean (SD), mg/dL	191.8 (38.45)	178.31 (38.16)	187.01 (35.81)	173.24 (36.77)	< 0.001
Triglycerides, mean (SD), mg/dL	167.65 (111.49)	129.88 (94.65)	135.19 (110.01)	102.68 (74.8)	< 0.001
Body-mass index, mean (SD), kg/m^2^	26.88 (4.45)	24.91 (4.12)	24.25 (3.32)	22.58 (3.37)	< 0.001
Hs-CRP, mean (SD), mg/dL	0.56 (0.95)	0.49 (1.06)	0.07 (0.04)	0.06 (0.04)	< 0.001
HbA1c, mean (SD), %	6.52 (1.37)	5.19 (0.27)	6.28 (1.26)	5.15 (0.27)	< 0.001
Sex, *n* (%)					0.002
Male	383 (50.7)	327 (47.9)	383 (49.6)	831 (43.7)	
Female	373 (49.3)	355 (52.1)	389 (50.4)	1072 (56.3)	
Regular exercise, *n* (%)					< 0.001
Yes	235 (31.1)	180 (26.4)	276 (35.8)	500 (26.3)	
No	521 (68.9)	502 (73.6)	496 (64.2)	1403 (73.7)	
Smoking, *n* (%)					< 0.001
Yes	194 (25.7)	141 (20.7)	170 (22)	366 (19.2)	
Quit	65 (8.6)	39 (5.7)	52 (6.7)	83 (4.4)	
No	497 (65.7)	502 (73.6)	550 (71.2)	1454 (76.4)	
Alcohol consumption, *n* (%)					0.110
Yes	253 (33.5)	244 (35.8)	276 (35.8)	729 (38.3)	
No	503 (66.5)	438 (64.2)	496 (64.2)	1174 (61.7)	
Heart disease, *n* (%)					< 0.001
Yes	76 (10.1)	35 (5.1)	75 (9.7)	100 (5.3)	
No	680 (89.9)	647 (94.9)	697 (90.3)	1803 (94.7)	
Stroke, *n* (%)					< 0.001
Yes	26 (3.4)	9 (1.3)	13 (1.7)	12 (0.6)	
No	730 (96.6)	673 (98.7)	759 (98.3)	1891 (99.4)	
Chronic infection or inflammation, *n* (%)					0.147
Yes	16 (2.12)	20 (2.93)	25 (3.24)	36 (1.89)	
No	740 (97.88)	662 (97.07)	747 (96.76)	1867 (98.11)	

*SD* standard deviation

^*^Analyzed using a Chi-squared test and Kruskal-Wallis H test

^†^The cutoff points for high levels of hs-CRP and HbA1c were based on the 66 percentile values: hs-CRP ≥0.15 mg/dL and Hba1c ≥5.60%

During the 8 years of follow-up, subjects with high levels of both hs-CRP and HbA1c (crude HR = 5.69; 95% CI = 3.08 ~ 10.51; *P* < 0.001), individuals with a high level of only hs-CRP (crude HR = 3.66; 95% CI = 1.86 ~ 7.19; *P* < 0.001), and those with a high level of HbA1c (crude HR = 6.85; 95% CI = 3.79 ~ 12.40; *P* < 0.001; Table 2) were associated with a higher risk of developing ACD than those with no high levels. However, after controlling for age, sex, SBP, DBP, glucose, total cholesterol, triglycerides, BMI, regular exercise, smoking status, alcohol consumption, heart disease, stroke, and chronic infection or inflammation, the results in Table 2 demonstrated that those with only a high level of hs-CRP had a higher risk of developing ACD (adjusted HR = 2.58; 95% CI = 1.29 ~ 5.17; *P* = 0.007), followed by the group with only a high level of HbA1c (adjusted HR = 2.52; 95% CI = 1.34 ~ 4.74; *P* = 0.004) and the groups with high levels of both hs-CRP or HbA1c (adjusted HR = 2.36; 95% CI = 1.20 ~ 4.62; *P* = 0.012; Table 2).

**Table 2:** Associations between the combined effects of high-sensitivity C-reactive protein (hs-CRP) and glycated hemoglobin (HbA1c) levels with the risk of dementia

Combined effect of hs-CRP and HbA1c	Numbers	Event	PYs	Crude model		Adjusted model ^†^	
				HR (95% CI)	*P* value ^*^	HR (95% CI)	*P* value ^*^
High levels of both hs-CRP and HbA1c	756	32	5850	5.69 (3.08 ~ 10.51)	< 0.001	2.36 (1.20 ~ 4.62)	0.012
High level of only hs-CRP	682	19	5408	3.66 (1.86 ~ 7.19)	< 0.001	2.58 (1.29 ~ 5.17)	0.007
High level of only HbA1c	772	40	6082	6.85 (3.79 ~ 12.40)	< 0.001	2.52 (1.34 ~ 4.74)	0.004
Non-high levels in both	1903	15	15,586	1.00		1.00	

*PYs* person-years, *HR* hazard ratio, *CI* confidence interval

^*^Analyzed using Cox proportional hazards regression analyses

^†^Adjusted for age, sex, systolic blood pressure, diastolic blood pressure, glucose, total cholesterol, triglycerides, body mass index, exercise status, smoking status, alcohol consumption, heart diseases, stroke, and chronic infection or inflammation

^‡^Cutoff points for a high level of hs-CRP and HbA1c were based on the 66th percentiles: hs-CRP ≥0.15 mg/dL and Hba1c ≥5.60%

Additionally, the results of the subgroup analysis presented inconsistent findings compared to the whole study sample. Among those without diabetes mellitus, groups with only a high level of hs-CRP (adjusted HR = 2.89; 95% CI = 1.27 ~ 6.56; *P =* 0.011) and HbA1c (adjusted HR = 2.27; 95% CI = 1.11 ~ 4.67; *P =* 0.026; Table 3) both had a greater risk of ACD, whereas those with high levels of both hs-CRP and HbA1c showed borderline significant results (*P =* 0.055). Nonetheless, Table 3 shows that only those in the group with a high level of inflammation, as measured by hs-CRP, presented a significant result for the development of ACD (adjusted HR = 11.33; 95% CI = 1.05 ~ 122.39; *P* = 0.046) in participants aged less than 65 years, but not in the group with high levels of both inflammation and hyperglycemia (*P* = 0.205) and the group with a high level of only HbA1c (*P* = 0.172). However, the adjusted hazard ratios for ACD risk were not significantly different among the four groups in those aged 65 years or older, and the same was seen for the secondary endpoints of Alzheimer’s disease and vascular dementia among the total sample (all *P >* 0.05).

**Table 3:** Subgroup analysis on the relationship between the combined effects of high-sensitivity C-reactive protein (hs-CRP) and glycated hemoglobin (HbA1c) with the risk of dementia, Alzheimer’s disease, and vascular dementia

Combined effect of hs-CRP and HbA1c	Numbers	Incidence rate per 10,000 PYs	Adjusted model ^†^	
			HR (95% CI)	*P-*value ^*^
Primary endpoints of dementia among those without diabetes mellitus (*n* = 3838)				
High levels of both hs-CRP and HbA1c	741	42.87	2.17 (0.98 ~ 4.77)	0.055
High level of only hs-CRP	569	33.26	2.89 (1.27 ~ 6.56)	0.011
High level of only HbA1c	898	50.12	2.27 (1.11 ~ 4.67)	0.026
Non-high levels in both	1630	7.48	1.00	
Primary endpoints of dementia among those aged less than 65 years ^#^ (*n* = 3481)				
High levels of both hs-CRP and HbA1c	667	3.70	5.75 (0.39 ~ 85.70)	0.205
High level of only hs-CRP	531	6.97	11.33 (1.05 ~ 122.39)	0.046
High level of only HbA1c	772	6.34	5.03 (0.50 ~ 51.09)	0.172
Non-high levels in both	1511	0.80	1.00	
Primary endpoints of dementia among those aged 65 years and older ^&^ (*n* = 632)				
High levels of both hs-CRP and HbA1c	105	172.71	1.21 (0.59 ~ 2.50)	0.599
High level of only hs-CRP	113	274.49	1.33 (0.77 ~ 2.31)	0.307
High level of only HbA1c	120	257.37	1.75 (0.97 ~ 3.16)	0.064
Non-high levels in both	294	194.98	1.00	
Secondary endpoints of Alzheimer’s disease among the total sample ^‡^ (*n* = 4113)				
High levels of both hs-CRP and HbA1c	756	11.83	5.3 (0.94 ~ 29.85)	0.059
High level of only hs-CRP	682	0.00	–	–
High level of only HbA1c	772	9.68	3.24 (0.58 ~ 18.03)	0.179
Non-high levels in both	1903	1.28	1.00	
Secondary endpoints of vascular dementia among the total sample ^‡^ (*n* = 4113)				
High levels of both hs-CRP and HbA1c	756	15.22	4.92 (0.97 ~ 24.87)	0.054
High level of only hs-CRP	682	5.49	2.89 (0.47 ~ 17.85)	0.254
High level of only HbA1c	772	14.55	4.17 (0.87 ~ 19.94)	0.074
Non-high levels in both	1903	1.28	1.00	

*PYs* person-years, *HR* hazard ratio, *CI* confidence interval

^*^Analyzed using Cox proportional hazards regression

^†^Adjusted for age, sex, systolic blood pressure, diastolic blood pressure, glucose, total cholesterol, triglycerides, body mass index, exercise status, smoking status, alcohol consumption, heart diseases, stroke, and chronic infection or inflammation

^¶^Cutoff points for high levels of hs-CRP and HbA1c were based on the 66th percentiles: hs-CRP ≥0.15 mg/dL and Hba1c ≥5.50%

^#^Cutoff points for high levels of hs-CRP and HbA1c were based on the 66th percentiles: hs-CRP ≥0.14 mg/dL and Hba1c ≥5.50%

^&^Cutoff points for high levels of hs-CRP and HbA1c were based on the 66th percentiles: hs-CRP ≥0.23 mg/dL and Hba1c ≥6.00%

^‡^Cutoff points for a high level of hs-CRP and HbA1c were based on the 66th percentiles: hs-CRP ≥0.15 mg/dL and Hba1c ≥5.60%

## Discussion

After 8 years of follow-up, the results of the current study revealed that high levels of inflammation (represented with hs-CRP) and hyperglycemia (represented with hbA1c) were risk factors that predict a higher risk of ACD. Moreover, the participants with high levels of only inflammation presented a significant ACD risk among adults aged less than 65 years, whereas no such relationships were found in elderly people.

The results of the present study found that subjects with only high levels of hs-CRP had a higher risk of ACD, followed by those with only high levels of HbA1c and their combination. These findings are similar to those of published studies [27–32]. Those with high levels of inflammation and glycation had at least 2.4-fold (range, 2.4 to 3.8) [27–29] and 1.9-fold (range, 1.9–2.9) [30–32] changes, respectively, in their cognitive impairment. These published results showed that the risk of dementia appears to be associated with higher hs-CRP levels as opposed to HbA1c levels, which is comparable to our findings. The major role of systemic inflammation in the development of type 2 diabetes mellitus may be considered as a possible pathogenesis [33]. In addition, the mechanisms underlying the relationship between inflammation or hyperglycemia and cognitive dysfunction might be explained by impaired endothelial function, [34, 35] which can result in cerebral white matter hyperintensities [36, 37]. The pathologic stimuli contributing to the response of endothelial cells resulted in the initiation of vascular compromise via breakdown of the blood-brain barrier and could lead to subsequent leukoaraiosis in the brain [38].

Several studies have examined the combined impact of inflammation and glycated hemoglobin on subsequent adverse consequences, such as severity of coronary artery disease, [20] cardiovascular events, [21] progression of carotid atherosclerosis, [22] and hyperglycemia [39]. To the best of our knowledge, this is the first study to assess the causal association of the combined effect of hs-CRP and HbA1c simultaneously with cognitive decline, although their combined effect presented a significant but non-multiplicative effect with regard to ACD risk. A negative association between hs-CRP and HbA1c might be a potential interpretation, in which hs-CRP levels might be influenced by multiple factors and cannot be explained by HbA1c alone [40]. Although contradictory to the results of prior studies, HbA1c levels increased when hs-CRP levels increased [33, 41].

With regard to the subgroup analysis shown in Table 3, the participants in the group with only a high level of hs-CRP had an increased risk of developing ACD among adults aged less than 65 years, which is consistent with the results of other studies. An adverse relationship between hs-CRP and incident Alzheimer’s disease was observed in adults aged between 60 and 70.5 years [27]. However, most studies have not examined the association between Hba1c and dementia under 65 years of age. Notably, there were no significant difference in ACD risk in the elderly population (age ≥ 65 years) among the four groups when using a cutoff point of 0.23 mg/dL for hs-CRP and ≥ 6.00% for Hba1c. The findings of this study are comparable to those of previous studies [32, 42]. There was not an adverse association between hs-CRP level and cognitive function in older women, [42] and in seniors aged greater than 70.6 years, an inverse association was found [27]. Moreover, those with HbA1c levels between 5.7 and 6.4% showed a higher but not significant risk of ACD, while an HbA1c level ≥ 7% presented an increased risk of incident ACD [32]. Although the small sample size and small number of events might result in insignificant findings, this study still provided adequate statistical power (> 80%) with which to elucidate the relationship between hs-CRP or HbA1c and cognitive decline among younger and elderly individuals. In addition, all of the diabetic individuals in the TwSHHH survey were reported to have type 2 DM. In order to test the consistency of the results, a subgroup analysis among those without DM was performed. The analysis presented findings comparable to those reported in the total sample. Therefore, the diagnosis of DM may not have influenced our main findings. In addition, treatment for DM or diabetes control might reduce the possibility of developing dementia.

This study had several strengths. First, to the best of our knowledge, this is the first study to investigate the combined effect of hs-CRP and HbA1c on the subsequent risk of ACD. Second, the present study used a nationwide population-based dataset, which increased the representativeness and generalizability of the study sample. Third, the subtypes of dementia, including Alzheimer’s disease and vascular dementia, were included in the subgroup analysis, whereas a lower incidence of cognitive impairment might result in non-significant results. Finally, a cohort study design was adopted in this study. Thus, a clear temporal causality was well established. Moreover, some potential limitations of the present study should be mentioned. First, the confounders regarding lifestyle factors were based on self-reporting instruments. Therefore, the possibility of selection and recall bias may have occurred, and the findings were limited. Second, the claims data from Taiwan’s NHIRD did not provide detailed information about the severity of cognitive decline. The ascertainment of diagnosed dementia might have led to an underestimation. Third, although this study did not define dementia by including the prescription of anti-dementia medications, subjects with dementia who had claims data of at least two confirmed visits were included to increase the validity of the diagnosis. Finally, the exposure assessments of inflammation and glycation were identified by a single screening, while the determination of the dynamic changes in hs-CRP and HbA1c could not be defined. In addition, exposure misclassification may occur.

## Conclusions

In summary, a higher risk of developing ACD was associated not only with high levels of inflammation but also with high levels of glycated hemoglobin during the 8-year follow-up period in a nationwide population-based cohort. In addition, high-sensitivity C-reactive protein and glycated hemoglobin A1c are useful prognostic markers for detecting cognitive dysfunction. Future studies should focus on the clinical implementation of hs-CRP or HbA1c to monitor cognitive deficits.

## Data Availability

The data described in the manuscript, code book, and analytic code will not be made available because the data source used in this study is managed by the Health and Welfare Data Science Center, Ministry of Health and Welfare (HWDC, MOHW), for which researchers need to submit an application to acquire it for scientific purposes; thus, these data were not publicly accessed.

## Abbreviations

ACD: all-cause dementia
DM: diabetes mellitus
TwSHHH: Taiwanese Survey on Prevalence of Hypertension, Hyperglycemia, and Hyperlipidemia
NHIRD: National Health Insurance Research Database
ICD-9-CM: International Classification of Diseases, Ninth Revision, Clinical Modification
AD: Alzheimer’s disease
IR: Insulin resistance
HR: Hazard ratio
CI: Confidence Interval
HWDC: Health and Welfare Data Science Center
MOHW: Ministry of Health and Welfare
SD: standard deviation
SBP: systolic blood pressure
DBP: diastolic blood pressure
BM: body mass index
